# USABILITY AND PSYCHOMETRIC PROPERTIES OF THE MOBITEC-GP APP FOR REAL-LIFE MOBILITY ASSESSMENT

**DOI:** 10.1093/geroni/igad104.0908

**Published:** 2023-12-21

**Authors:** Eleftheria Giannouli, Eun-Kyeong Kim, Cheng Fu, Robert Weibel, Alexandros Sofios, Arno Schmidt-Trucksäss, Andreas Zeller, Timo Hinrichs

**Affiliations:** ETH Zurich, Zurich, Zurich, Switzerland; Luxembourg Institute of Socio-Economic Research, Luxembourg, Diekirch, Luxembourg; University of Zurich, Zurich, Zurich, Switzerland; University of Zurich, Zurich, Zurich, Switzerland; University of Zurich, Zurich, Zurich, Switzerland; University of Basel, Basel, Basel-Stadt, Switzerland; University of Basel, Basel, Basel-Stadt, Switzerland; University of Basel, Basel, Basel-Stadt, Switzerland

## Abstract

Modern technologies such as the Global Navigation Satellite System and accelerometers which are integrated in every smartphone enable tracking mobility patterns over time, which in turn enables early detection of mobility impairments. Aim of the MOBITEC-GP project was to develop a mobile app which measures walking speed and spatial mobility under everyday conditions. Reliability and validity of the application was tested first (Study 1). The applicability in everyday life and the acceptance by older adults (Study 2) was also investigated. In study 1, participants underwent several supervised walking speed assessments as well as a 1-week life-space assessment during two assessment sessions 9 days apart. Fifty-seven older adults (47.4% male, mean age= 75.3 years) were included in the study. The app showed moderate to excellent test-retest reliability and validity of walking speed measurements of 50 meters and above and of most 1-week life-space parameters, including life-space area, time spent out-of-home, and action range. Study 2 revealed a good usability of the app (mean System Usability Scale score=77.18) among older adults (N=60 with ≥2 chronic conditions) for a 1-week usage of the app including both, a 1-week life space assessment and at least one gait speed measurement in a 30-minute stroll. The MOBITEC-GP app is a reliable, valid and usable tool for the assessment of real-life walking speed (at distances of 50 metres and above) and life-space parameters of older adults. Future studies should look into technical issues more systematically in order to avoid invalid measurements.

